# Rural household income mobility in Ethiopia: Dimensions, drivers and policy

**DOI:** 10.1371/journal.pone.0284987

**Published:** 2023-09-14

**Authors:** Yalfal Temesgen Tigabu, Mengistu Ketema Aredo, Alelign Ademe

**Affiliations:** 1 School of Agricultural Economics and Agribusiness, Haramaya University, Dire Dawa, Ethiopia; 2 Ethiopian Economic Association, Addis Ababa, Ethiopia; 3 University of Eswatini, Kwaluseni, Lesotho; Szechenyi Istvan University: Szechenyi Istvan Egyetem, HUNGARY

## Abstract

Welfare dynamics studies are useful in understanding how individuals, families, society, and a country are organised. For the last two decades, Ethiopia’s economic reports on income disparity, poverty, and other welfare metrics have been hopeful and controversial. It is crucial to understand how rural households of various income levels perform over time and income mobility. Income mobility can be observed as a change in position over time between two income vectors, with some climbing and others sliding down and changing places at various rates. This study, therefore, explored the rural households’ income mobility in Ethiopia using three waves of the Living Standards Measurement Study-Integrated Survey on Agriculture (LSMS-ISA) collected from 2011 to 2016. The Shorrocks rigidity index, transition probability matrix, Fields, and Ok methods were employed to analyse the relative and absolute income mobility. The logit model with conditional fixed effect was used to assess the drivers of individual households’ income mobility and the multinomial logit model with conditional fixed effect as an alternative model. Based on the finding of this study, it is suggested to implement different policies targeting income growth to shorten mobility gaps and address factors contributing to downward income mobility in rural households in Ethiopia are necessary.

## 1. Introduction

The World Bank (WB) and the African Development Bank (ADB) data show that African countries’ gross domestic product (GDP) reached 3.4% in 2019 and 3.9% in 2020. Africa’s real GDP was projected to increase by 3.4% in 2021 after contracting by 2.1% in 2020. This projected recovery from the worst recession in more than half a century will be supported by a resumption of tourism, a rebound in food prices, and the rollback of pandemic-induced restrictions [1].

According to the World Bank’s Ethiopia Poverty Assessment, the national poverty rate has dropped, falling from 30% of the population in 2011 to 24% in 2016. The research notes, however, that the poorest 10% of the population has seen no income rise since 2005. In 2016 the disadvantaged were poorer than those in 2005 [1]. Conversely, the Gini coefficient, measuring income inequality, increased since 1995, from 0.29 in 1995 to 0.3 in 2010/2011 and 0.33 in 2015/2016.

Studies recommended investigating the relevance of how people are moving along the income distribution using welfare indicators, such as income, consumption, and asset. Such investigation is essential during the courses of the economic process [2, 3]. This is vital because a society’s progress is often gauged by how much people’s well-being or living standards improved and how much socio-economic deprivation was reduced over time. This allows for identifying priorities that will put the needs of the people first while discussing the challenges societies’ encounter [4, 5].

The extent to which households move across various economic, income mobility, is a central issue in various public policy discussions. Income mobility is a ladder representing income distribution. Some move up from one step to another at various rates, whereas others slide down [6]. Understanding how income evolves is often observed as examining equality of opportunity [7, 8]. This is because various income mobility levels, such as upward movers and downward movers, call for a mix of policies.

Extensive empirical studies where most of the literature was on advanced economies and little on developing countries; in Ethiopia, no evidence exists of income mobility. The current study aimed to provide empirical evidence on rural households’ income mobility and their determinants in Ethiopia. The study specifically intended to evaluate income mobile households and identify income mobility upward and downward drivers.

## 2. Empirical review on income mobility

In policy debates, dynamic societies are often seen as desirable since they offer a fair chance of moving up the income ladder [9–11]. To assess the extent to which societies provide individual members such opportunities to escape their origins, a common approach has been to measure the degree of income mobility in society with income mobility itself often calculated as the lack of correlation between past income and present income [2, 12, 13].

Mobility can be viewed either as a time-dependent or movement measure of income change [13]. Empirical studies using the time-dependent concept assume an influence of past income on income changes [9, 14–16]. On the other hand, the movement measure strand of studies considers a change in the rank or position of an agent between two periods [17]. Another dimension is an analysis of income mobility within generations (intra-generational) or between generations (intergenerational).

In developed countries with a few in developing countries that are mostly based in Latin America [15, 17]. A study in Chile point out that upward mobility was enhanced by a change from unemployment to employment, higher education, urban residence, being married and female headship but it was inversely influenced by male headship and the number of children [17]. The same factors (apart from gender and marital status) influenced downward 40 mobility except they switched signs. In the case of gender, male-headed households were less likely to move either up or down while marital status was only significant for upward movement.

Most of the empirical evidence assembled in the survey reveals that current knowledge is derived to a considerable extent from developed countries, where there has no tradition of collecting panel data in developing countries in general, eastern African countries specifically. [18] attempt to analyse income mobility in Uganda using income quintiles and find higher mobility at the bottom of the welfare distribution with more than half the households located in the lowest quintile in 2009–10 moving up the welfare distribution in 2010–11 and 40 percent of the households in the highest quintile in 2009–10 moving down the welfare distribution in 2010–11.

Economic mobility in Kenya and Egypt using a three-period panel and showed that low-income persistence for the poor and uneducated may be evidence of cumulative disadvantage and the possible existence of poverty traps [19–21]. As expected, higher education seems to eliminate the low-income persistence for these vulnerable groups and allow convergence of incomes towards their average. [22] studied the impact of the non-farm economy on consumption growth in Ethiopia using Ethiopia rural households survey data collected in six rounds over ten years, from 1994 to 2004.

## 3. Research methods

### 3.1. Study area

The study is located in nine regional states in Ethiopia where rural and small-town households were the main targets. Currently, based on official population statistics reported in 2007, Ethiopia has a total population of 73.7 million [23]. However, in 2019 Ethiopia has a population of approximately 112.08 million based on 2015’s estimate of 98.9 million which makes it the 14th most populous country in the world with a total of 1,104,300 km^2^ (426,372.6137 miles square) surface area and population density of 83 people per square mile (214/square mile), which ranks 123rd in the world [1].

**Fig 1:**
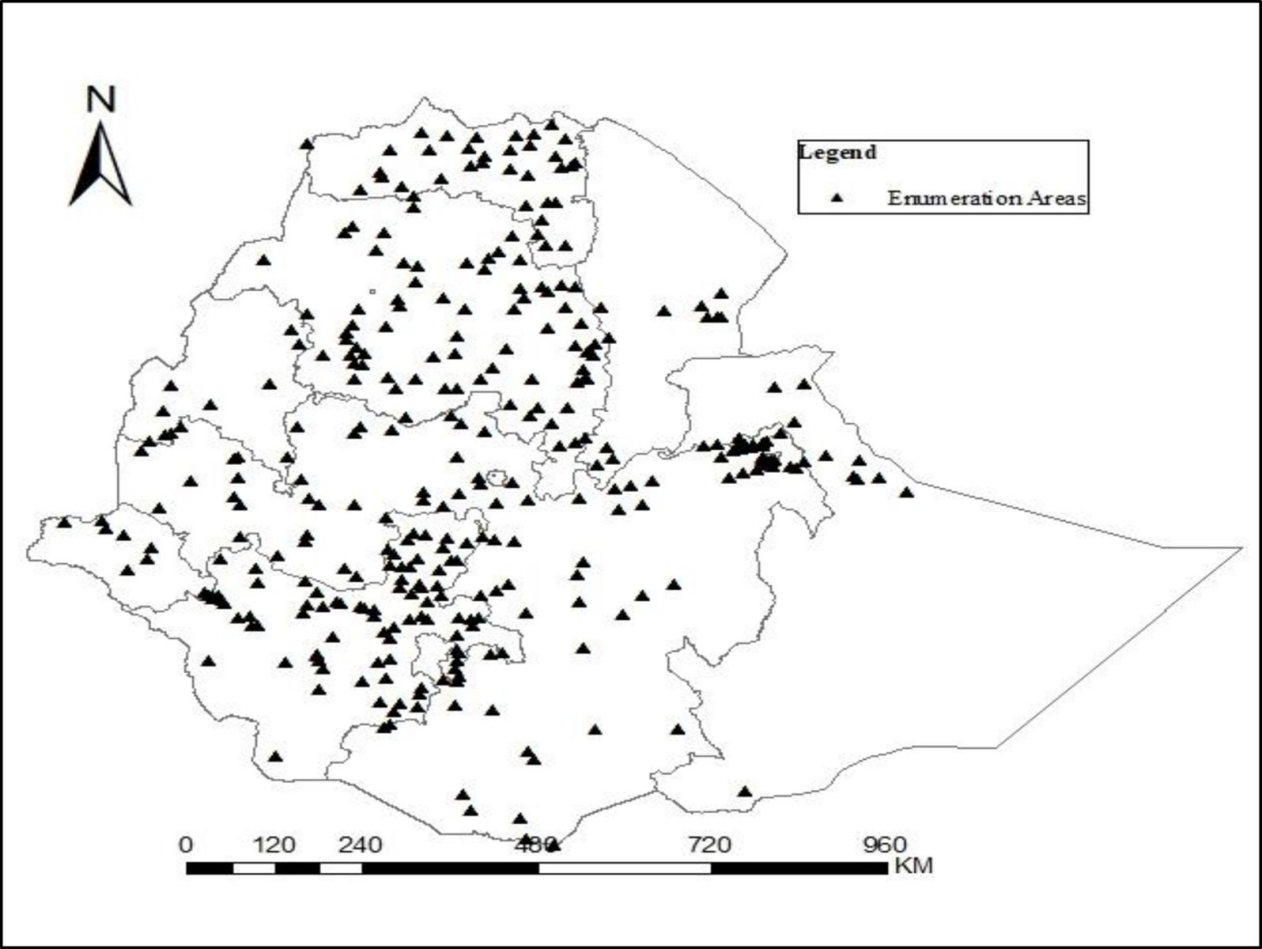
The geographical location of enumeration areas of the study (EAS).

The agricultural sector is the cornerstone of Ethiopia’s economy with approximately three-quarters of the economically active population contributing to engagement in agricultural production activities. The sector contributes 37 percent to GDP, one of the highest shares in sub-Saharan Africa, as well as 83.9 percent to exports. Moreover, the sector employs around 72 percent of the total population. The country’s topographic diversity results in varied farming systems, enabling crop and livestock production in the highlands and agro-pastoralist in the lowlands [22, 24–27]. Fig 1 below shows the study area in general and the data collection areas specifically.

### 3.2. Data sources and type

This study used panel data collected by the World Bank in collaboration with Ethiopia Central Statistics Agency (CSA) as the Living Standard Measurement Survey-Integrated Agricultural Survey (LSMS-ISA) collected from 2011 to 2016. The survey has three rounds collected in 2011/2, 2013/4, and 2015/6 as the first wave, second wave, and third wave, respectively. The panel dataset is a national wide survey collecting multistage probability household samples.

First, the study domains were identified (regions, urban/rural) employing a stratified random design. Second, enumeration areas (EAs) were selected with probability proportional to size. Finally, the primary sampling units (PSU) were geographically defined area units selected with probability proportional to size based on the last population census in the country.

3,969 households from wave one, 5,262 households from wave two and 4,954 households from wave three were interviewed, with a 6.8 attrition rate for rural households. The study was based on rural households and large towns. Data were, therefore, collected only in the second and third waves. Those samples from urban were automatically excluded from the analysis. The study further restricted the sample size because of missing information in the consumption data. These indicate where households reported a purchase price, but there is no purchase record or lacking conversion factors (prices) for certain items the households consumed and households with zero total consumption (Table 1). Finally, this study exploited 3,239 households at each wave and 9,717 household observations for the analysis.

**Table 1:** Test of equality of means of socio-economic and institutional characteristics across consumption quintiles.

Variables	Overall	Consumption Quintile					
		1st	2nd	3^rd^	4th	5th	F-stat
Land size (ha)	1.35	1.17	1.37	1.49	1.63	1.12	2.22*
	(6.43)	(2.88)	(3.31)	(3.31)	(8.44)	(3.40)	
Cultivated land (ha)	1.391	1.18	1.38	1.55	1.67	1.16	2.34*
	(6.30)	(2.83)	(3.24)	(10.45)	(7.85)	(2.18)	
Livestock Holding (TLU)	2.73	2.19	2.7	2.89	2.90	2.88	11.88***
	(3.57)	(2.59)	(3.72)	(3.53)	(3.62)	(4.11)	
Distance to main road (km)	16.41	16.37	16.37	16.52	16.82	15.99	0.38
	(21.98)	(18.93)	(21.10)	(22.68)	(23.55)	(22.92)	
Distance to P. Center (km)	40.49	40.89	39.51	40.54	40.07	41.33	0.66
	(33.66)	(28.31)	(30.02)	(34.06)	(36.04)	(37.75)	
Distance from nearest Market (km)	66.37	84.26	65.28	61.76	61.96	60.62	73.47***
	(50.55)	(59.70)	(46.66)	(46.96)	(948.41)	(47.01)	

Note

*** p < .01

** p < .05

* p < .1 and figures presented are mean (standard devetion)

Source: author’s calculation using ESS data 2011/12, 2013/13 and 2015/16 waves

### 3.3. Method of data analysis

#### 3.3.1. Shorrocks rigidity index

Most income dynamics studies focused on how the existence of mobility is measured in economies. This study employed the Shorrocks rigidity index to identify income mobility in relative terms [7, 28, 29]. Shorrocks index, one of the single-stage indexes, was constructed using the Gini of the average income between the periods with the weighted average of the Gini in each period as follows:

$$R=\frac{G(x+y+z)}{\mu_{x}G_{x}+\mu_{y}G_{y}+\mu_{z}G_{yz}/\mu_{x}+\mu_{y+}\mu_{z}}$$ (1)

Where R is the rigidity index, Gx refers to the Gini coefficient, and μ_x_ refers to mean income in the first period; Gy refers to the Gini coefficient, and μ_y_ refers to mean income in the final period. The result of the rigidity index is interpreted as meaning no mobility, whereas zero would indicate perfect mobility. The larger the value rigidity index means, the lesser mobility and the larger the permanent component of inequality measures; the smaller the value of the rigidity index means, the higher mobility and smaller the permanent inequality measure component.

#### 3.3.2. Transition probability matrix

Cases exist where a single-stage index, such as the Shorrocks index, may not capture a more disaggregated mobility. The Shorrocks rigidity index may reach no mobility if all income is increased at a constant proportion factor, only capturing the variation in income share and ranks.

Here, a two-stage index was suggested. This study, therefore, employed a transition probability matrix to analyse the existence of income mobility in absolute terms. The transition probability matrix was constructed by dividing it into endogenously determined income/consumption groups of equal sizes (quintile in our case). This matrix captures the growth dimension of income dynamics where immobility, upward mobility ratio, and downward mobility are described.

This transition matrix can be interpreted as households experiencing a change in income/consumption over three waves, from lowest to higher or contrary wise, called ‘mobiles’. Those who did not experience change are called ‘immobile’. From mobiles, those who experience change from lower-income/consumption groups to higher-income/consumption groups are called ‘upward income mobiles’. Those who experience change from higher-income/consumption groups to lower-income/consumption groups are called ‘downward income mobiles’.

#### 3.3.3. Determinants of rural household income mobility in Ethiopia

Binomial logit with a fixed-effect model was applied to analyse the determinants of household income mobility with a robustness check. A fixed-effect multinomial logit model was employed as an alternative model. This model used a separate analysis of upward and downward income mobility on the households’ relative income distribution using quintiles observed between 2011 to 2016. The quintiles were computed using population-weighted adult equivalent household consumption. For the upward income mobility, households were analysed either moving up or staying in the quintile. The same is true for downward income mobility; therefore, the study estimated the two models using the fixed-effect logit specification:

$$P(Y_{it}=1|x_{it},a_{it})=\Lambda (x_{it}\beta +a_{i})$$ (2)

Where; ^(x_it_β +a_i)_ is a logistic function, x_it_ is a vector of time-varying variables, a_i_ is an unobserved household effect. The model treats a_i_ to be estimated along with β. The advantage of the model is that there is no need to assume the distribution of a_it_/ x_it;_ that is, the unobserved heterogeneity may be correlated with the explanatory variables. The fixed-effect multinomial logit model was applied as a robustness check of the main result and an alternative model. [30] developed by [31], originated this model.

The model allows unobserved heterogeneity to be correlated with an observed independent variable, relating to assuming any unobserved heterogeneity is independent of the observed explanatory variable [31]. If the characteristics related to income mobility are similar or common for all households in the analysis of household income mobility, there is no need to control a_ij_ because a_ij_ = a_j._ In another ward round, if household characteristics interact with the income mobility categories, not control for a_ij_ cause the estimate to be inconsistent. This study assumed that the unobserved household’s characteristic if dissimilar for each income category and the model is specified as follows:

$$Y_{itj}=a_{itj}+x_{it}\beta_{j}+\mu_{itj}$$ (3)

Where Y_itj_ is the probability of households I at time t to income mobility category j, the outcome variable is the income mobility category, Y_i_ where j = 1…j represents three income mobility categories: (1) downward income mobility (j = 1), (2) no mobility or immobility (j = 2), and (3) upward income mobility (j = 3). X_it_ and β_j_ are a vector of explanatory variable and predicted coefficient, respectively; μ_itj_ is an error term. It is assumed that the error term is independent and distributed identically over time, and in households with mean zero and variance $\sigma_{u}^{2}$.

## 4. Results and discussion

### 4.1. Sampled households’ characteristics

Certain socio-economic characteristics contributed to upward and downward income mobility. These characteristics include gender, age, education, and household characteristics, such as education in the household, household members, and the number of dependent and working members. Table 2 provides a summary of statistics on household characteristics. In the sample household, 24% were female-headed, indicating most sample households were male-headed, and the mean age of the household is 46, with 14 and 100 years of minimum and maximum age, respectively.

**Table 2:** Determinants of upward income mobility.

Upward Mobility	OR	St.Err.	t-value	P-value	[95% Conf Interval]		Sig
Sex of the household Head	1.882	0.928	0.68	0.496	-1.187	2.451	
Age of the household Head	0.736	0.029	-10.47	0.000	-0.365	-0.25	***
Education Status	0.886	0.371	-0.33	0.745	-0.849	0.607	
Total Household size	0.041	0.296	-10.77	0.000	-3.771	-2.609	***
Land size	1.357	0.132	2.31	0.021	0.047	0.563	**
Livestock holding	1.156	0.060	2.42	0.016	0.027	0.263	**
Credit Use	1.000	0.000	0.93	0.350	0.000	0.000	
Distance to major road	1.082	0.089	0.89	0.374	-0.095	0.252	
Distance to nearest market	0.978	0.117	-0.19	0.848	-0.251	0.207	
Nonfarm Income	0.999	4.45e	-0.70	0.485	0.000	5.62e-06	
Farm Income	0.999	9.91e	-6.07	0.000	0.000	0.000	***
Food consumption	1.000	0.000	6.04	0.000	0.000	0.000	***
Non-Food consumption	1.000	0.000	0.31	0.757	0.000	0.000	
Exposure to shocks	0.577	0.189	-2.91	0.004	-0.920	-0.18	***
Assets	1.251	0.085	2.63	0.008	0.057	0.391	***
Extension contact	1.040	0.252	0.16	0.875	-0.453	0.533	
Irrigation Use	1.205	0.434	0.43	0.667	-0.663	1.037	
Pseudo r-squared	0.782						
LR chi2 (17)	1499.984						
Prob > chi2	0.000						
Akaike crit. (AIC)	449.875						
Bayesian crit. (BIC)	550.591						

Sources: Author’s calculation using ESS data 2011/12, 2013/13 and 2015/16 waves

*** p < .01

** p < .05

* p < .1.

Considering the whole family, the mean age in the household was 26. Thirty-nine per cent of the sampled household heads were illiterate. The education level in the household was two years of schooling. The intra-household dynamics of education may, therefore, provide a diverse representation of the function of education. The sampled households also held six members, the household with a 1.3% dependency ratio, which can be observed from two sides: the availability of labour and expenditure sides. The results also indicated that the mean number of female adults (1.4) is slightly greater than male adults (1.3) in the households.

In addition to the overall summary of each characteristic of the households, the statistics also have a report within and between summaries. The overall and within summary are based on the total number of households (N) observed in the three waves whereas the between statistics are based on household observation (n) at each wave (Table 3).

**Table 3:** Demographic characteristics of the sampled households.

Variable		Mean	Std. Dev.	Min	Max	Observations
Sex of the Household head	Overall	0.24359	0.42927	0	1	N = 9717
	Between		0.42417	0	1	n = 3239
	Within		0.06627	-0.42	0.911	T = 3
Age of the Household head	Overall	46.3099	15.4156	14	100	N = 9491
	Between		15.2720	15.67	100	n = 3238
	Within		3.23155	15.81	92.64	T-bar = 2.9313
Mean age in the household	Overall	25.9422	12.4458	6.75	97	N = 9715
	Between		11.523	10.85	91.33	n = 3239
	Within		4.70339	-17.50	64.81	T-bar = 2.9998
Household head Literacy	Overall	0.39302	0.48845	0	1	N = 9717
	Between		0.43365	0	1	n = 3239
	Within		0.22488	-0.27	1.06	T = 3
Education level in the Household	Overall	1.93269	1.42068	0	35	N = 9717
	Between		1.09534	0	19.67	n = 3239
	Within		0.90487	-13.73	23.93	T = 3
Household Size	Overall	5.55922	2.52063	1	18	N = 9717
	Between		2.38420	1	15.67	n = 3239
	Within		0.81872	-1.77	11.56	T = 3
Total Male Adults	Overall	1.32592	1.04138	0	9	N = 9717
	Between		0.92992	0	8.33	n = 3239
	Within		0.46895	-2.01	3.99	T = 3
Total Female Adults	Overall	1.41391	0.87737	0	8	N = 9717
	Between		0.76102	0	5.67	n = 3239
	Within		0.43675	-1.25	3.75	T = 3
Dependency Ratio	Overall	1.33378	1.08227	0	11	N = 9374
	Between		0.86870	0	6	n = 3181
	Within		0.65351	-2.49	8.23	T-bar = 2.946

Sources: Author’s calculation using ESS data 2011/12, 2013/13 and 2015/16 waves

### 4.2. Socio-economic and institutional characteristics of the sampled households

The socio-economics and institutional characteristics are important factors in household income mobility. These include total land holding, cultivated land size, livestock ownership, and infrastructure access. These socio-economics and institutional characteristics were tested across the consumption expenditure quintiles to determine the statistical difference among consumption quintiles. The test was also conducted to determine which consumption quintiles differ from others. According to Table 4, considering socio-economic and access to infrastructures, land size, cultivated land, livestock holding, distance to the nearest market, and zone capital have a statistically significant mean difference among consumption quintiles.

**Table 4:** Determinants of downward income mobility.

Downward Mobility	OR	St.Err.	t-value	p-value	[95% Conf Interval]	
Sex of the household Head	0.000	22.905	-0.47	0.636	-55.738	34.046
Age of the household Head	1.496	0.079	5.13	0.000	0.249	0.557
Education Status	0.896	0.903	-0.12	0.904	-1.879	1.660
Total Household size	917.45	1.252	5.45	0.000	4.367	9.276
Land size	0.319	0.492	-2.33	0.020	-2.107	-0.18
Livestock holding	0.693	0.170	-2.15	0.031	-0.700	-0.033
Credit Use	1.001	0.000	0.53	0.599	0.000	0.000
Distance to major road	0.724	0.205	-1.57	0.116	-0.725	0.080
Distance to nearest market	1.136	0.404	0.32	0.751	-0.664	0.920
Nonfarm Income	1.000	0.001	0.97	0.332	0.000	0.001
Farm Income	1.000	0.001	2.59	0.010	0.000	0.000
Food consumption	0.999	0.001	-5.73	0.000	-0.001	-0.001
Non-Food consumption	0.999	0.001	-1.42	0.155	0.000	0.000
Exposure to shocks	1.949	0.444	1.50	0.133	-0.203	1.538
Assets	0.542	0.194	-3.16	0.002	-0.993	-0.233
Extension contact	0.567	0.555	-1.02	0.306	-1.656	0.520
Irrigation Use	5.642	1.230	1.41	0.160	-0.681	4.141
Pseudo r-squared	0.926					
Prob > chi2	0.000					
Chi-square	1045.819					
Bayesian crit. (BIC)	209.748					

Sources: Author’s calculation using ESS data 2011/12, 2013/13 and 2015/16 waves

*** p < .01, ** p < .05, * p < .1

The mean land size of the sampled household was 1.17, 1.37, 1.49, 1.63 and 1.12 hectares in the first; second, third, fourth, and fifth consumption quintiles, respectively. This is comparable with the national average of about 1.17 hectares [22]. The Tukey post hoc test indicates a statistically significant difference in the mean of land size and cultivated land between the fourth and fifth consumption quintiles.

The sampled households owned 2.19, 2.7, 2.89, 2.9 and 2.88 TLU on average in the first, second, third, fourth, and fifth consumption quintiles, respectively. Households owned 2.73 TLU on average; this is lower than the national average. The post hoc test indicated a statistically significant difference in the mean of livestock holding between the first and the other consumption quintiles.

Access to institutional services, such as the market, is crucial in households’ income mobility. A household member must travel to a major market on average 84.3, 65.28, 61.76, 61.96 and 60.62 km in the first, second, third, fourth, and fifth consumption quintiles, respectively. The post hoc test indicated a statistically significant difference in the mean of livestock holding between the first and other consumption quintiles.

### 4.3. Consumption and income heterogeneity

The sampled households exhibited heterogeneity in selected categories, such as gender, education, credit use, main livelihoods, and vulnerability to shocks. As indicated in Table 5, the mean differences concerning consumption, income and assets are compared across selected categories. The result indicated that the gender group has a statistically mean difference concerning food consumption, non-food consumption, and farm income. Male-headed households consumed more food (ETB 5618) and non-food (ETB 1204) than female-headed households. This is because male-headed households earn ETB 10887 (t-stat = 3.33) more farm income than female-headed households.

**Table 5:** Mean comparison of consumption, income and asset across selected categories.

Group	Variable	Sex of the Household Head	Literacy Household Head	Credit Use	Crop based Livelihood	Livestock based Livelihood	Shocks (Overall shocks)
Cons	Food	5618.8**	-3109.18**	-369.46	-2164.66**	-4326.14**	5618.81**
		(319.54)	(332.34)	(698.87)	(372.20)	(421.56)	(-1482.53)
	Non-food	1204.9**	-1964.52**	-741.51**	797.26**	124.62	151.76
		(114.91)	(130.09)	(256.56)	(146.88)	(145.5)	(138.7)
	Total	6860.0**	-5272.42**	-1164.06	-1258.8**	-4180.3**	-1329.54**
		(368.30)	(382.74)	(794.32)	(441.3)	(480.5)	(377.33)
Income	Farm	10887.9**	4004.68	157.89	-10803.06**	-17196.1**	-10803.06
		(3271.66)	(4177.74)	(5124.5)	(3099.53)	(2890.14)	(5818.28)
	Nonfarm	701.83	-595.25	-232.56	8658.10	2865.52	-3909.44
		(2840.9)	(3405.48)	(2396.6)	(5323.15)	(4094.71)	(3732.2)
	Total	11589.68	3409.42	-74.68	-2144.96	-14330.58	-11911.9**
		(6912.41)	(6075.77)	(7520.9)	(7473.41)	(7439.75)	(5940.28)

Note

*** p < .01

** p < .05

* p < .1, and the figures presented are mean (standard deviation); mean differences by each wave are given in S1 Table

Source: Author’s calculation using ESS data 2011/12, 2013/13, and 2015/16 waves

The result also revealed that the literacy level group (categorised as literate and non-literate) has a statistically significant difference in mean food consumption, non-food consumption, durable asset, housing characteristic, and farm equipment. Literate households consumed more food (ETB 3109) and non-food (ETB 1964) items. Concerning assets, literate has better durable assets, housing characteristics, and farm equipment, leading to higher consumption. A statistically significant difference exists in mean food consumption, housing characteristic, and farm equipment, considering credit use.

Regarding the main livelihood of the households, crop-based livelihood has a statistically significant difference in the mean of food consumption, non-food consumption, farm income, durable assets, housing characteristic, and farm equipment. Household as main crop producers consumed ETB 2164 (t-stat = -9.33) and ETE 797 (t-stat = 5.43) more food and non-food items. As expected, this difference comes from the farm income, which is the sale of crops. Concerning shocks, vulnerable households have a statistically significant difference in mean food consumption, durable assets, housing characteristic, and farm equipment. This household consumed ETB 5618 more food items. This holds a more durable asset, housing characteristics, and farm equipment.

### 4.4. Income mobility

#### 4.4.1. Relative income mobility

The study employed the Shorrocks rigidity index to estimate the relative income mobility of households based on adult equivalent consumption with per capita income for comparison. Table 6 presents a summary of the results of Shorrocks’ rigidity. The result indicated that income data estimates are higher than consumption data. This is because of two reasons; first, consumption smoothing makes expenditure less erratic; second, respondents’ behaviour reduces inequality. For instance, in the case of expenditure, the poor reported well whereas the rich usually forget it; in the case of income, the rich have a predicted and more stable income than the poor. As a result, the poor understate their income [32, 33].

**Table 6:** Shorrock’s rigidity index using income and expenditure, 2011–2016.

Gini Coefficient	Income-based		Consumption-based	
	Per capita	Total	Per adult equivalent	Total
Gini 2011	0.4116	0.427	0.75575	0.77502
Gini 2013	0.3375	0.36529	0.54975	0.54526
Gini 2016	0.3446	0.36384	0.76837	0.77897
Average Gini	0.3636	0.38409	0.69129	0.69975
Income/consumption 2011	5059.7	19430.37	2609.03	13445.68
Income/consumption 2013	5071.1	19538.02	2617.11	13639.9
Income/consumption 2016	5640.6	21537	5587.91	35226.19
Shorrock Rigidity Index	0.9993	0.9987	0.9703	9626

Sources: Author’s calculation using ESS data 2011/12, 2013/13, and 2015/16 waves

The result also indicated that Ethiopia’s rural households have a Shorrocks rigidity index of 0.97, implying a higher rate of income mobility in income and expenditure data. This result follows a study in Egypt and South Africa, establishing that Shorrocks’ rigidity index was 0.95 for income and 0.934 for consumption. The conclusion indicates that Egypt is characterised by high mobility [4, 15].

#### 4.4.2. Absolute income mobility

The result of relative income mobility is significant; however, it is essential to evaluate mobility further, turning it into a transition matrix for a further disaggregated observation. The relative income measurement did not show the difference in income because of the increase of the proportion factor, which only captures the variation of income shares or ranks. Tables 7 and 8 present the transition matrix for expenditure and income. The transition matrix first allocated households into income/expenditure groups, income quintile in our case where quintiles numbered from 1 for poorest to 5 for richest, then examines the mobility among these income/expenditure quintiles groups.

**Table 7:** Transition matrix by quintile using expenditure (Percentages), 2011–2016.

Wave 1	Wave 2						Wave 2	Wave 3					
		1	2	3	4	5			1	2	3	4	5
	1	51.46	20.97	13.40	7.570	6.6		1	46.36	24.03	14.57	10.39	4.65
	2	25.59	23.96	24.68	16.94	8.83		2	24.36	24.87	24.19	17.67	8.92
	3	18.33	22.75	22.27	2.43	14.22		3	16.11	22.74	21.84	23.49	15.81
	4	11.77	16.28	23.26	28.63	20.06		4	7.14	18.54	25.23	26.29	22.8
	5	4.83	10.14	18.51	21.93	44.58		5	6.53	12.48	16.69	24.24	40.06
Total		19.91	18.0	20.50	20.31	21.27			19.76	20.35	20.41	20.56	18.93

Sources: Author’s calculation using ESS data 2011/12, 2013/13, and 2015/16 waves

Note: the estimate is based on annual adult equivalent consumption

**Table 8:** Transition matrix by quintile using income (Percentages), 2011–2016.

Wave 1	Wave 2						Wave 2	Wave 3					
		1	2	3	4	5			1	2	3	4	5
	1	21.8	23.99	21.47	17.44	15.32		1	36.70	21.1	14.9	14.5	12.8
	2	12.5	25.34	28.03	22.03	12.12		2	20.27	28.5	21.4	17.8	12.1
	3	7.76	16.30	23.91	31.37	20.65		3	12.69	20.2	22.4	26.3	18.4
	4	7.19	6.130	18.60	31.71	36.36		4	9.74	10.5	18.3	27.6	33.8
	5	10.9	5.750	15.65	17.57	50.16		5	9.54	5.47	9.96	19.4	55.9
Total		13.5	18.43	22.63	23.46	22.01			15.93	16.3	17.5	21.9	28.3

Sources: Author’s calculation using ESS data 2011/12, 2013/13, and 2015/16 waves

Note: the estimate is based on annual income per capita

It can be observed that 45% of the household in the richest quintile in 2011 remained there in 2013, and another 22% moved down just one quintile; 52% of those who began in the poorest quintile were still there three years later. Another 21% had moved up just one quintile. Similarly, 40% of the household in the richest quintile in 2013 remained there in 2016, and another 24% moved down just one quintile.

Likewise, 46% of those who began in the poorest quintile remained there three years later; another 24% moved up one quintile. This indicated less mobility in the bottom and top quintiles than in the distribution. This can also be confirmed using the number of elements established in the diagonal section of the transition matric.

The number of elements in the transition matrix established in the right of the diagonal section is slightly less than the element in the left, meaning expecting a slighter income mobility experience indicating there is less mobility in the top and bottom quintile than in the distribution. This is because the bottom (top) quintiles can only stay in the same quintile the reason persistence in that group is high (Table 7).

As a robustness assessment, the study further analysed the transition rate among waves (2011–2013, 2011–2016) using income data. The result indicated that 22% of the household in the richest quintile in 2011 remained there in 2013, and another 23% moved down one quintile. Likewise, 22% of those who began in the poorest quintile remained there three years later; another 4% moved up one quintile. Similarly, 28% of the household in the richest quintile in 2013 remained there in 2016. Another 22% moved down one quintile. Likewise, 37% of those who began in the poorest quintile remained there three years later; another 21% moved up one quintile. The matrix for income and expenditure are, therefore, remarkably similar (Table 8).

#### 4.4.3. Income mobility determinants

The analysis of determinants of income mobility was conducted using binomial logistic with fixed-effect regressions separately for the determinants of upward and downward income mobility with the same households treated differently. Fixed-effect models are important devices to control unobserved heterogeneity related to observed covariates, assess the causality between income mobility and explanatory variables, and reduce omitted variable bias [31, 34, 35].

Considering the aforementioned, this study conducted a diagnostic test to select the appropriate model. The Breusch-Pagan Lagrange multiplier (LM) test distinguished between fixed-effect and pooled OLS. The result confirmed considerable evidence (Prob > chi2 = 0.00) to accept the fixed-effect model. After selecting the fixed-effect model, the study proceeded to the other test to select between fixed and random effect models using the Hausman specification test. The Hausman specification test is based on the null hypothesis that if the time-invariant individual effects are uncorrelated with the regressor, the random effect will be selected.

The result from the Hausman specification test in S2 Table indicates that a random-effects model of the initial hypothesis of the individual-level effects is resoundingly rejected (P-value = 0.00). The fixed effect is an appropriate model. The result of determinants of upward and downward income mobility are discussed alongside. Tables 2 and 4 indicate that upward and downward income mobility is influenced by the age of the household head, total family size, land holding, livestock holding, farm income, food consumption, exposure to shock, and asset holding. This result follows the literature and is symmetrical between upward and downward mobility [17, 29].

The study established that the household heads’ age is less inclined to upward income mobility and more inclined to experience downward income mobility. This is because as age increases, the participation in non-farm activities and sharing of properties, such as land, will increase [1, 6, 22, 26, 36–39].

The household size was established with a negative effect at a 1% significance level in the upward income mobility; however, the effect is positive for the downward income mobility. The possibility of being in downward income mobility is, therefore, high for a household of a generous size [25, 27, 36].

Landholding positively influenced upward income mobility as opposed to downward income mobility. As the land size increased by one unit, the odds of the household experiencing upward income mobility rose by 1.357. The possibility of being in upward income mobility is, therefore, high for those households with higher land size [16].

Livestock holding positively determined upward income mobility. As expected, as the household livestock holdings increased by one unit, the odds of the households’ upward income mobility rose by 1.156. This is because the livestock increased the probability of participating in commercialisation while diversifying the household income [1, 16].

The household farm income negatively and significantly affects income mobility; being in farm income activities decreases the odds of upward income mobility by 0.999. This implies that the household participation in farm income harms off-farm/non-farm income; therefore, the total gain decreases [40, 41].

Expenditure on food items positively determined upward income mobility. The odds of household upward mobility rise by 1% as the household expenditure on food items increases. This means that the probability of the household experiencing upward income increases as the food items’ expenditure share increased compared to other expenditures [39].

Asset holding also has a significant and positive effect on upward income mobility and negatively downward income mobility. This implies that households with more asset holding are more inclined to move up and less inclined to move down in income [24, 30, 37, 42, 43]. Exposure to shocks has a negative and significant effect on upward income mobility. As exposure to household shock increases by one unit, the odds of a household’s upward income mobility will fall by 0.577.

Besides the binomial logit with a fixed-effect model, the study presents the finding from a robustness check applying the fixed-effect multinomial logit model as an alternative method (S1 Table). The fixed-effect multinomial logit model extends standard multinomial logit, multinomial logit with random effect, and fixed-effect logit [30, 31]. The main purpose of additional analysis of the fixed-effect multinomial logit model is to examine how certain the estimate behaves when the dependent variable has three categories. The results from the multinomial logit with a fixed-effect model follow the binomial approach (S1 Table).

## 5. Conclusions

A recent estimate of income inequality, poverty, and other welfare indicators in Ethiopia signified improvements. These crucial economic development indicators cannot respond to: Why are the rich getting richer and the poor getting poorer? Who are the winners and losers during the economic process? To answer these questions, an income mobility analysis, tracking the households’ income, must be conducted.

The descriptive statistics of the study indicated that most sample households were male-headed (76%) aged 46. Most of the sampled household heads (61%) were literate; at least they could read and write. The sampled households also have a large family size of six persons on average, with a dependency ratio of 1.30. Mean comparisons of socio-economic and institutional characteristics across consumption quintiles indicated that the mean land holding of the sampled households followed the national average with a statistically significant mean of landholding for the last two consumption quintiles.

The average livestock holding of the sampled households in TLU was lower than the national average. The mean comparison for consumption, income, and asset across selected categories indicated that male-headed and educated households have higher expenditures. Shorrocks’ rigidity index and the transition probability matrix indicated the sampled households experienced slighter income mobility, demonstrating relative and absolute income mobility in Ethiopia.

The fixed-effect logit model result indicated that a household’s income mobility was statistically influenced by the age of the household head, total family size, total land holding, total livestock holding, farm income, food consumption, exposure to shock, and asset holding. The result from the alternative model follows the main model. The results were symmetrical between upward and downward mobility.

Policy and intervention should consider the structure of age, supporting and strengthening the family planning programmes and incentives that may motivate rural households to build assets. Fragmented land-use patterns and the land rental markets, and the importance of livestock husbandry should be considered in the well-being of households.

## Supporting information

S1 Table Determinants of income mobility: Fixed-effects multinomial logistic. (DOCX)

S2 Table Huasman result for fixed and random effect model selection for income mobility (first, second and third models). (DOCX)
