# Supplementary material for: Rural household income mobility in Ethiopia: Dimensions, drivers and policy
Source: PLoS One. 2023 Sep 14;18(9):e0284987. doi: 10.1371/journal.pone.0284987 (PMC10501615; doi:10.1371/journal.pone.0284987)
Supplement: S1 Table — (DOCX) [file pone.0284987.s001.docx]

**S1 Table. Determinants of income mobility: fixed-effects multinomial logistic.**

| Variables | Downward Mobility | | | Upward mobility | | |
| --- | --- | --- | --- | --- | --- | --- |
|  | Coef. | Std.Err. | P>z | Coef. | Std.Err. | P>z |
| Gender of the household head | -12.497 | 3.038 | 0.000 | -1.651 | 0.944 | 0.080 |
| Age of the household head | -0.076 | 0.131 | 0.560 | -0.479 | 0.088 | 0.000 |
| Education status | 0.039 | 1.082 | 0.971 | 0.149 | 0.559 | 0.790 |
| Total household size | 4.251 | 1.064 | 0.000 | -2.571 | 0.645 | 0.000 |
| land size | -0.855 | 0.577 | 0.138 | 0.288 | 0.227 | 0.204 |
| Livestock holding | -0.253 | 0.120 | 0.035 | 0.114 | 0.055 | 0.038 |
| Credit use | 0.000 | 0.000 | 0.309 | 0.000 | 0.000 | 0.724 |
| Distance to major road | -0.024 | 0.379 | 0.950 | 0.299 | 0.351 | 0.395 |
| Distance to nearest market | 0.006 | 0.361 | 0.986 | -0.122 | 0.142 | 0.393 |
| Non-farm income | 0.000 | 0.000 | 0.200 | -0.000 | 0.000 | 0.986 |
| Farm income | 0.000 | 0.000 | 0.918 | -0.000 | 0.000 | 0.001 |
| Food consumption | -0.000 | 0.000 | 0.016 | -0.000 | 0.000 | 0.061 |
| Non-food consumption | -0.001 | 0.000 | 0.000 | -0.000 | 0.000 | 0.779 |
| Exposure to shocks | 0.006 | 0.625 | 0.992 | -0.661 | 0.379 | 0.081 |
| Assets | -0.524 | 0.243 | 0.031 | 0.089 | 0.199 | 0.655 |
| Extension contact | -0.640 | 0.627 | 0.307 | -0.072 | 0.307 | 0.814 |
| Irrigation use | -2.233 | 1.396 | 0.110 | -0.502 | 0.665 | 0.450 |
| Wald chi2 (34) = 188.14 Prob > chi2 = 0.0000 Log pseudo likelihood = -109.51872  Pseudo R2 = 0.8857 | | | | | | |

Note: *** p<.01, ** p<.05, * p<.1, base category is immobility

Sources: Author’s calculation using ESS data 2011/12, 2013/13 and 2015/16 waves
