# Supplementary material for: Rural household income mobility in Ethiopia: Dimensions, drivers and policy
Source: PLoS One. 2023 Sep 14;18(9):e0284987. doi: 10.1371/journal.pone.0284987 (PMC10501615; doi:10.1371/journal.pone.0284987)
Supplement: S2 Table — (DOCX) [file pone.0284987.s002.docx]

**S2 Table. Huasman result for fixed and random effect model selection for income mobility (first, second and third models).**

| Variables | Coefficients | | Difference (b-B) | Sqrt (diag(V_b-V_B))  S.E. |
| --- | --- | --- | --- | --- |
|  | Fixed effect (b) | Random effect (B) |  |  |
| Sex of the household Head | .6320799 | -.1913203 | .8234002 | .9236805 |
| Age of the household Head | -.3071219 | -.00519 | -.3019319 | .0292499 |
| Education Status | -.1209522 | -.1711301 | .050178 | .3641948 |
| Total Household size | -3.190204 | -.2190756 | -2.971128 | .2958558 |
| land size | .3049797 | .1272007 | .177779 | .1290106 |
| Livestock holding | .1450959 | .0816779 | .063418 | .0580173 |
| Credit Use | .0000148 | 1.60e-06 | .0000132 | .0000156 |
| Distance to major road | .0787726 | -.001629 | .0804016 | .0885494 |
| Distance to nearest market | -.022307 | -.0013509 | -.020956 | .1167426 |
| Nonfarm Income | -3.11e-06 | -6.31e-07 | -2.48e-06 | 4.41e-06 |
| Farm Income | -.0000602 | -.0000241 | -.0000361 | 9.64e-06 |
| Food consumption | 3.22e-06 | -5.33e-06 | 8.55e-06 | 9.33e-06 |
| Non-Food consumption | .0000768 | .0000353 | .0000415 | .0000123 |
| Exposure to shocks | -.5499566 | -.4284231 | -.1215335 | .1770202 |
| Assets | .2239528 | .0151393 | .2088134 | .0839185 |
| Extension contact | .0396052 | .0594715 | -.0198663 | .2414617 |
| Irrigation Use | .1868119 | .194266 | -.0074541 | .4213202 |
| **Huasman (1978) specification test**   \| Chi-square test value =185.457  P-value =0.000 \| \| --- \| | | | | |
