# Supplementary material for: Rural household income mobility in Ethiopia: Dimensions, drivers and policy
Source: PLoS One. 2023 Sep 14;18(9):e0284987. doi: 10.1371/journal.pone.0284987 (PMC10501615; doi:10.1371/journal.pone.0284987)
Supplement: Attachment — Submitted filename: Response to Reviewers.docx [file pone.0284987.s003.docx]

**Response to Reviewers**

**Reviewer: 1**

The manuscript is technically solid, the data obtained by the authors supports the investigation and its conclusions, and thus the study exhibits novel research findings. What was particularly applauded was the careful introduction of methods and the reasons behind using them.

The paper's format is logical, beginning with an overview of the problem and progressing through the methods and sources of data collecting. The studies are carried out to a high technical degree and are sufficiently detailed within the publication, demonstrating that the analysis was carried out appropriately and systematically. The authors have included all data supporting their findings in their publication in tables, along with explanations for those tables. Additionally, the conclusion is given in the appropriate manner, the evidence supports the conclusions.
The research complies with all applicable ethical and research integrity standards, and the work adheres to appropriate reporting criteria and community standards for data access.
The only (absolutely minor) issue with the most recent version of the paper is one mistake i found (on page 13, where mean number of male adults is 1.3, not 1.4.), so a thorough second check is required with the data, but my overall recommendation is to publish the paper following a check.

Response: I appreciate you seeing this. We sincerely apologize for this error. We have fixed that, and the sampled households’ characteristics section of the main documents has correct been corrected.

**Reviewer: 2**

Rural Household Income Mobility in Ethiopia: Dimensions, Drivers and Policy paper is an important, gap-filling work, as no evidence exist of income mobility in Ethiopia. It aims to provide empirical evidence on rural households' income mobility and their determinants in the horn of Africa; consequently the study provides a sound basis for policy recommendations.

The chosen analytical tools complement each other well, and their combination is appropriate. The description - presentation, justification - of each analytical method is clear, coherent, and well structured.

I have some additional comments for minor corrections:

- The numbers should be uniform, either written out in letters or as numbers. In this the manuscript is inconsistent, see e.g. 2.1 Data: 3rd paragraph 1st line

- Decimal points should also be used consistently in number division, see e.g. 2.1 Data: 3rd paragraph last sentence.

- In 3.1 Sampled households' characteristics it says: "The results also indicated that the mean number of female adults (1.4) is slightly greater than male adults (1.4) in the households." But the referred values are equal. Write out more decimal places to support the claim.

- In the conclusion it would be worth highlighting the most disadvantaged and making specific recommendations concerning them.

Response: (1) thank you for noting this. As you indicated, we changed the specific comment that was made, and we used that as a cue to change the entire article such that all reports used consistent numbers or letters.

Response: (2) thank you for noting this and we take it for the whole article to be consistent in decimal points.

Response: (3) The concert rose on “3.1 sampled households' characteristics” were also raised by reviewer one, we have fixed that, the error were occurred during minimizing the digits. Mean number of female adults (1.4) is slightly greater than male adults (1.3)

Response: (4) The suggestion given regarding specific conclusions and recommendation for the most disadvantaged group accepted and we appreciate your insightful suggestions.
