# Supplementary material for: Rural household income mobility in Ethiopia: Dimensions, drivers and policy
Source: PLoS One. 2023 Sep 14;18(9):e0284987. doi: 10.1371/journal.pone.0284987 (PMC10501615; doi:10.1371/journal.pone.0284987)
Supplement: Peer review history

# Peer review: Decision Letter 0

**DOI:** 10.1371/journal.pone.0284987.r001

**Authors:** László Vasa

26 Jan 2023

PONE-D-22-28184Rural Household Income Mobility in Ethiopia: Dimensions, Drivers and PolicyPLOS ONE

Dear Dr. Tigabu,

Thank you for submitting your manuscript to PLOS ONE. After careful consideration, we feel that it has merit but does not fully meet PLOS ONE’s publication criteria as it currently stands. Therefore, we invite you to submit a revised version of the manuscript that addresses the points raised during the review process.

Please submit your revised manuscript by Mar 12 2023 11:59PM. If you will need more time than this to complete your revisions, please reply to this message or contact the journal office at plosone@plos.org. When you're ready to submit your revision, log on to https://www.editorialmanager.com/pone/ and select the 'Submissions Needing Revision' folder to locate your manuscript file.

Please include the following items when submitting your revised manuscript:

- A rebuttal letter that responds to each point raised by the academic editor and reviewer(s). You should upload this letter as a separate file labeled 'Response to Reviewers'.
- A marked-up copy of your manuscript that highlights changes made to the original version. You should upload this as a separate file labeled 'Revised Manuscript with Track Changes'.
- An unmarked version of your revised paper without tracked changes. You should upload this as a separate file labeled 'Manuscript'.

If you would like to make changes to your financial disclosure, please include your updated statement in your cover letter. Guidelines for resubmitting your figure files are available below the reviewer comments at the end of this letter.

If applicable, we recommend that you deposit your laboratory protocols in protocols.io to enhance the reproducibility of your results. Protocols.io assigns your protocol its own identifier (DOI) so that it can be cited independently in the future. For instructions see: https://journals.plos.org/plosone/s/submission-guidelines#loc-laboratory-protocols. Additionally, PLOS ONE offers an option for publishing peer-reviewed Lab Protocol articles, which describe protocols hosted on protocols.io. Read more information on sharing protocols at https://plos.org/protocols?utm_medium=editorial-email&utm_source=authorletters&utm_campaign=protocols.

We look forward to receiving your revised manuscript.

Kind regards,

László Vasa, PhD

Academic Editor

PLOS ONE

Journal Requirements:

When submitting your revision, we need you to address these additional requirements.

1. Please ensure that your manuscript meets PLOS ONE's style requirements, including those for file naming. The PLOS ONE style templates can be found at 

https://journals.plos.org/plosone/s/file?id=wjVg/PLOSOne_formatting_sample_main_body.pdf and 

https://journals.plos.org/plosone/s/file?id=ba62/PLOSOne_formatting_sample_title_authors_affiliations.pdf

2. Thank you for stating the following financial disclosure: 

6687

At this time, please address the following queries:

a) Please clarify the sources of funding (financial or material support) for your study. List the grants or organizations that supported your study, including funding received from your institution. 

b) State what role the funders took in the study. If the funders had no role in your study, please state: “The funders had no role in study design, data collection and analysis, decision to publish, or preparation of the manuscript.”

c) If any authors received a salary from any of your funders, please state which authors and which funders.

d) If you did not receive any funding for this study, please state: “The authors received no specific funding for this work.”

Please include your amended statements within your cover letter; we will change the online submission form on your behalf.

3. PLOS requires an ORCID iD for the corresponding author in Editorial Manager on papers submitted after December 6th, 2016. Please ensure that you have an ORCID iD and that it is validated in Editorial Manager. To do this, go to ‘Update my Information’ (in the upper left-hand corner of the main menu), and click on the Fetch/Validate link next to the ORCID field. This will take you to the ORCID site and allow you to create a new iD or authenticate a pre-existing iD in Editorial Manager. Please see the following video for instructions on linking an ORCID iD to your Editorial Manager account: https://www.youtube.com/watch?v=_xcclfuvtxQ

4. We note that you have referenced (ie. Bewick et al. [5]) which has currently not yet been accepted for publication. Please remove this from your References and amend this to state in the body of your manuscript: (ie “Bewick et al. [Unpublished]”) as detailed online in our guide for authors

http://journals.plos.org/plosone/s/submission-guidelines#loc-reference-style

[Note: HTML markup is below. Please do not edit.]

Reviewers' comments:

Reviewer's Responses to Questions

**Comments to the Author**

1. Is the manuscript technically sound, and do the data support the conclusions?

The manuscript must describe a technically sound piece of scientific research with data that supports the conclusions. Experiments must have been conducted rigorously, with appropriate controls, replication, and sample sizes. The conclusions must be drawn appropriately based on the data presented. 

Reviewer #1: Yes

Reviewer #2: Yes

Reviewer #3: Partly

**********

2. Has the statistical analysis been performed appropriately and rigorously? 

Reviewer #1: Yes

Reviewer #2: Yes

Reviewer #3: Yes

**********

3. Have the authors made all data underlying the findings in their manuscript fully available?

The PLOS Data policy requires authors to make all data underlying the findings described in their manuscript fully available without restriction, with rare exception (please refer to the Data Availability Statement in the manuscript PDF file). The data should be provided as part of the manuscript or its supporting information, or deposited to a public repository. For example, in addition to summary statistics, the data points behind means, medians and variance measures should be available. If there are restrictions on publicly sharing data—e.g. participant privacy or use of data from a third party—those must be specified.

Reviewer #1: Yes

Reviewer #2: Yes

Reviewer #3: Yes

**********

4. Is the manuscript presented in an intelligible fashion and written in standard English?

PLOS ONE does not copyedit accepted manuscripts, so the language in submitted articles must be clear, correct, and unambiguous. Any typographical or grammatical errors should be corrected at revision, so please note any specific errors here.

Reviewer #1: Yes

Reviewer #2: Yes

Reviewer #3: Yes

**********

5. Review Comments to the Author

Please use the space provided to explain your answers to the questions above. You may also include additional comments for the author, including concerns about dual publication, research ethics, or publication ethics. (Please upload your review as an attachment if it exceeds 20,000 characters)

Reviewer #1: The manuscript is technically solid, the data obtained by the authors supports the investigation and its conclusions, and thus the study exhibits novel research findings. What was particularly applauded was the careful introduction of methods and the reasons behind using them.

The paper's format is logical, beginning with an overview of the problem and progressing through the methods and sources of data collecting. The studies are carried out to a high technical degree and are sufficiently detailed within the publication, demonstrating that the analysis was carried out appropriately and systematically. The authors have included all data supporting their findings in their publication in tables, along with explanations for those tables. Additionally, the conclusion is given in the appropriate manner, the evidence supports the conclusions.

The research complies with all applicable ethical and research integrity standards, and the work adheres to appropriate reporting criteria and community standards for data access.

The only (absolutely minor) issue with the most recent version of the paper is one mistake i found (on page 13, where mean number of male adults is 1.3, not 1.4.), so a thorough second check is required with the data, but my overall recommendation is to publish the paper following a check.

Reviewer #2: Rural Household Income Mobility in Ethiopia: Dimensions, Drivers and Policy paper is an important, gap-filling work, as no evidence exist of income mobility in Ethiopia. It aims to provide empirical evidence on rural households' income mobility and their determinants in the horn of Africa, consequently the study provides a sound basis for policy recommendations.

The chosen analytical tools complement each other well, and their combination is appropriate. The description - presentation, justification - of each analytical method is clear, coherent, and well structured.

I have some additional comments for minor corrections:

- The numbers should be uniform, either written out in letters or as numbers. In this the manuscript is inconsistent, see e.g. 2.1 Data: 3rd paragraph 1st line

- Decimal points should also be used consistently in number division, see e.g. 2.1 Data: 3rd paragraph last sentence.

- In 3.1 Sampled households' characteristics it says: "The results also indicated that the mean number of female adults (1.4) is slightly greater than male adults (1.4) in the households." But the referred values are equal. Write out more decimal places to support the claim.

- In the conclusion it would be worth highlighting the most disadvantaged and making specific recommendations concerning them.

Reviewer #3: The paper is focusing on a timely and actual topic of household income mobility in Africa, specifically in Ethiopia. The paper's focus, context, research methodology can be regarded as original and, in my opinion, it can contribute to the existing knowledge. The authors selected the appropriate method and methodological toolset and made the research well.

However, in the paper, in fact, we can't see any literature review. Without this, the paper can't be regarded as scientific writing. I recommend to write a 'classical' literature review which is analysing, processing the most relevant international sources in a critical and comparative way.

**********

6. PLOS authors have the option to publish the peer review history of their article (what does this mean?). If published, this will include your full peer review and any attached files.

If you choose “no”, your identity will remain anonymous but your review may still be made public.

**Do you want your identity to be public for this peer review?** For information about this choice, including consent withdrawal, please see our Privacy Policy.

Reviewer #1: No

Reviewer #2: No

Reviewer #3: No

**********

[NOTE: If reviewer comments were submitted as an attachment file, they will be attached to this email and accessible via the submission site. Please log into your account, locate the manuscript record, and check for the action link "View Attachments". If this link does not appear, there are no attachment files.]

While revising your submission, please upload your figure files to the Preflight Analysis and Conversion Engine (PACE) digital diagnostic tool, https://pacev2.apexcovantage.com/. PACE helps ensure that figures meet PLOS requirements. To use PACE, you must first register as a user. Registration is free. Then, login and navigate to the UPLOAD tab, where you will find detailed instructions on how to use the tool. If you encounter any issues or have any questions when using PACE, please email PLOS at figures@plos.org. Please note that Supporting Information files do not need this step.

# Author comment: Author response to Decision Letter 0

**DOI:** 10.1371/journal.pone.0284987.r002

12 Mar 2023

Reviewer: 1

The manuscript is technically solid, the data obtained by the authors supports the investigation and its conclusions, and thus the study exhibits novel research findings. What was particularly applauded was the careful introduction of methods and the reasons behind using them.

The paper's format is logical, beginning with an overview of the problem and progressing through the methods and sources of data collecting. The studies are carried out to a high technical degree and are sufficiently detailed within the publication, demonstrating that the analysis was carried out appropriately and systematically. The authors have included all data supporting their findings in their publication in tables, along with explanations for those tables. Additionally, the conclusion is given in the appropriate manner, the evidence supports the conclusions.

The research complies with all applicable ethical and research integrity standards, and the work adheres to appropriate reporting criteria and community standards for data access.

The only (absolutely minor) issue with the most recent version of the paper is one mistake i found (on page 13, where mean number of male adults is 1.3, not 1.4.), so a thorough second check is required with the data, but my overall recommendation is to publish the paper following a check.

Response: I appreciate you seeing this. We sincerely apologize for this error. We have fixed that, and the sampled households’ characteristics section of the main documents has correct been corrected.

Reviewer: 2

Rural Household Income Mobility in Ethiopia: Dimensions, Drivers and Policy paper is an important, gap-filling work, as no evidence exist of income mobility in Ethiopia. It aims to provide empirical evidence on rural households' income mobility and their determinants in the horn of Africa; consequently the study provides a sound basis for policy recommendations.

The chosen analytical tools complement each other well, and their combination is appropriate. The description - presentation, justification - of each analytical method is clear, coherent, and well structured.

I have some additional comments for minor corrections:

- The numbers should be uniform, either written out in letters or as numbers. In this the manuscript is inconsistent, see e.g. 2.1 Data: 3rd paragraph 1st line

- Decimal points should also be used consistently in number division, see e.g. 2.1 Data: 3rd paragraph last sentence.

- In 3.1 Sampled households' characteristics it says: "The results also indicated that the mean number of female adults (1.4) is slightly greater than male adults (1.4) in the households." But the referred values are equal. Write out more decimal places to support the claim.

- In the conclusion it would be worth highlighting the most disadvantaged and making specific recommendations concerning them.

Response: (1) thank you for noting this. As you indicated, we changed the specific comment that was made, and we used that as a cue to change the entire article such that all reports used consistent numbers or letters.

Response: (2) thank you for noting this and we take it for the whole article to be consistent in decimal points.

Response: (3) The concert rose on “3.1 sampled households' characteristics” were also raised by reviewer one, we have fixed that, the error were occurred during minimizing the digits. Mean number of female adults (1.4) is slightly greater than male adults (1.3)

Response: (4) The suggestion given regarding specific conclusions and recommendation for the most disadvantaged group accepted and we appreciate your insightful suggestions.

Reviewer: 3

The paper is focusing on a timely and actual topic of household income mobility in Africa, specifically in Ethiopia. The paper's focus, context, research methodology can be regarded as original and, in my opinion, it can contribute to the existing knowledge. The authors selected the appropriate method and methodological toolset and made the research well.

However, in the paper, in fact, we can't see any literature review. Without this, the paper can't be regarded as scientific writing. I recommend to write a 'classical' literature review which is analyzing, processing the most relevant international sources in a critical and comparative way.

Response: We appreciate your wise suggestions. We both agree that further details regarding the literature review have to be included. The idea you presented has been bolstered by the addition of the most pertinent international sources to the literature section.

Academic Editor

When submitting your revision, we need you to address these additional requirements.

1. Please ensure that your manuscript meets PLOS ONE's style requirements, including those for file naming. 

2. Thank you for stating the following financial disclosure: 

6687

At this time, please address the following queries:

a) Please clarify the sources of funding (financial or material support) for your study. List the grants or organizations that supported your study, including funding received from your institution. 

b) State what role the funders took in the study. If the funders had no role in your study, please state: “The funders had no role in study design, data collection and analysis, decision to publish, or preparation of the manuscript.”

c) If any authors received a salary from any of your funders, please state which authors and which funders.

d) If you did not receive any funding for this study, please state: “The authors received no specific funding for this work.”

Please include your amended statements within your cover letter; we will change the online submission form on your behalf.

3. PLOS requires an ORCID iD for the corresponding author in Editorial Manager on papers submitted after December 6th, 2016. Please ensure that you have an ORCID iD and that it is validated in Editorial Manager. To do this, go to ‘Update my Information’ (in the upper left-hand corner of the main menu), and click on the Fetch/Validate link next to the ORCID field. This will take you to the ORCID site and allow you to create a new iD or authenticate a pre-existing iD in Editorial Manager. Please see the following video for instructions on linking an ORCID iD to your Editorial Manager account: 

4. We note that you have referenced (ie. Bewick et al. [5]) which has currently not yet been accepted for publication. Please remove this from your References and amend this to state in the body of your manuscript: (ie “Bewick et al. [Unpublished]”) as detailed online in our guide for authors

Response: (1) We appreciate your feedback, and we made every effort to ensure that the manuscript adhered to PLOS ONE's style guidelines.

Response: (2a) The "Africa Economic Research Consortium" provided funding for this study as part of a PhD Research Award. Unfortunately, the authors did not receive any particular support for this project.

Response: (2b) the funders for this study “Africa Economic Research Consortium” had no role in study design, data collection and analysis, decision to publish, or preparation of the manuscript.”

Response: (2c) we (all) author of this study did not receive a salary from any of funders in relation to this study.

Response: (2d) “The authors received no specific funding for this work.”

Response: (3) My ORCID iD: https://orcid.org/ 0000-0002-9260-181X

Response: (4) We appreciate you catching this. The reference that was not yet accepted for publication was removed from the paper, and the references were changed to reflect your suggestions.

Attachment Submitted filename: Response to Reviewers.docx Click here for additional data file.

# Peer review: Decision Letter 1

**DOI:** 10.1371/journal.pone.0284987.r003

**Authors:** László Vasa

13 Apr 2023

Rural Household Income Mobility in Ethiopia: Dimensions, Drivers and Policy

PONE-D-22-28184R1

Dear Dr. Tigabu,

We’re pleased to inform you that your manuscript has been judged scientifically suitable for publication and will be formally accepted for publication once it meets all outstanding technical requirements.

Within one week, you’ll receive an e-mail detailing the required amendments. When these have been addressed, you’ll receive a formal acceptance letter and your manuscript will be scheduled for publication.

An invoice for payment will follow shortly after the formal acceptance. To ensure an efficient process, please log into Editorial Manager at http://www.editorialmanager.com/pone/, click the 'Update My Information' link at the top of the page, and double check that your user information is up-to-date. If you have any billing related questions, please contact our Author Billing department directly at authorbilling@plos.org.

If your institution or institutions have a press office, please notify them about your upcoming paper to help maximize its impact. If they’ll be preparing press materials, please inform our press team as soon as possible -- no later than 48 hours after receiving the formal acceptance. Your manuscript will remain under strict press embargo until 2 pm Eastern Time on the date of publication. For more information, please contact onepress@plos.org.

Kind regards,

Prof. László Vasa, PhD

Academic Editor

PLOS ONE

Additional Editor Comments (optional):

Reviewers' comments:

Reviewer's Responses to Questions

**Comments to the Author**

1. If the authors have adequately addressed your comments raised in a previous round of review and you feel that this manuscript is now acceptable for publication, you may indicate that here to bypass the “Comments to the Author” section, enter your conflict of interest statement in the “Confidential to Editor” section, and submit your "Accept" recommendation.

Reviewer #1: All comments have been addressed

Reviewer #2: All comments have been addressed

**********

2. Is the manuscript technically sound, and do the data support the conclusions?

The manuscript must describe a technically sound piece of scientific research with data that supports the conclusions. Experiments must have been conducted rigorously, with appropriate controls, replication, and sample sizes. The conclusions must be drawn appropriately based on the data presented. 

Reviewer #1: Yes

Reviewer #2: Yes

**********

3. Has the statistical analysis been performed appropriately and rigorously? 

Reviewer #1: Yes

Reviewer #2: Yes

**********

4. Have the authors made all data underlying the findings in their manuscript fully available?

The PLOS Data policy requires authors to make all data underlying the findings described in their manuscript fully available without restriction, with rare exception (please refer to the Data Availability Statement in the manuscript PDF file). The data should be provided as part of the manuscript or its supporting information, or deposited to a public repository. For example, in addition to summary statistics, the data points behind means, medians and variance measures should be available. If there are restrictions on publicly sharing data—e.g. participant privacy or use of data from a third party—those must be specified.

Reviewer #1: Yes

Reviewer #2: Yes

**********

5. Is the manuscript presented in an intelligible fashion and written in standard English?

PLOS ONE does not copyedit accepted manuscripts, so the language in submitted articles must be clear, correct, and unambiguous. Any typographical or grammatical errors should be corrected at revision, so please note any specific errors here.

Reviewer #1: Yes

Reviewer #2: Yes

**********

6. Review Comments to the Author

Please use the space provided to explain your answers to the questions above. You may also include additional comments for the author, including concerns about dual publication, research ethics, or publication ethics. (Please upload your review as an attachment if it exceeds 20,000 characters)

Reviewer #1: Based on the new version provided, it appears that the authors have done a huge job in conducting their research and addressing the feedback provided by the reviewers.

I noted in my review that the manuscript is technically solid and that the authors' data supports the investigation and its conclusions, also praised the authors for their careful introduction of methods and the reasons behind using them. They have changed those issues i recommended.

Reviewer #2 stated that the paper is an important and gap-filling work that provides empirical evidence on rural households' income mobility and their determinants in Ethiopia. However, the reviewer suggested some minor corrections, such as ensuring uniformity in numbers and decimal points. According to the updated version, the author addressed these comments.

Finally, Reviewer #3 noted that the authors' research methodology can be regarded as original and that it can contribute to existing knowledge. However, the reviewer recommended that the authors include a literature review that analyzes and processes the most relevant international sources in a critical and comparative way, as this is a necessary component of scientific writing. The authors did provide a lit review in the new version.

Overall, it appears that the authors have done a great job in addressing the feedback provided by the reviewers and conducting their research. Their work on rural household income mobility in Ethiopia is important and contributes to existing knowledge on the topic.

Reviewer #2: All comments have been processed by the author. As a review I was unable to examine the dual publication issue, however the present manuscript is of high quality.

**********

7. PLOS authors have the option to publish the peer review history of their article (what does this mean?). If published, this will include your full peer review and any attached files.

If you choose “no”, your identity will remain anonymous but your review may still be made public.

**Do you want your identity to be public for this peer review?** For information about this choice, including consent withdrawal, please see our Privacy Policy.

Reviewer #1: No

Reviewer #2: No

**********

# Editor report: Acceptance letter

**DOI:** 10.1371/journal.pone.0284987.r004

**Authors:** László Vasa

12 May 2023

PONE-D-22-28184R1 

Rural household income mobility in Ethiopia: Dimensions, drivers and policy 

Dear Dr. Tigabu:

I'm pleased to inform you that your manuscript has been deemed suitable for publication in PLOS ONE. Congratulations! Your manuscript is now with our production department. 

If your institution or institutions have a press office, please let them know about your upcoming paper now to help maximize its impact. If they'll be preparing press materials, please inform our press team within the next 48 hours. Your manuscript will remain under strict press embargo until 2 pm Eastern Time on the date of publication. For more information please contact onepress@plos.org.

If we can help with anything else, please email us at plosone@plos.org. 

Thank you for submitting your work to PLOS ONE and supporting open access. 

Kind regards, 

PLOS ONE Editorial Office Staff

on behalf of

Prof. Dr. László Vasa 

Academic Editor

PLOS ONE
